# Prognostic Value and Longitudinal Dynamics of Multigene Circulating Tumor DNA in Advanced Pancreatic Cancer Treated with 5-Fluorouracil-Based Chemotherapy

**DOI:** 10.3390/ijms27198668

**Published:** 2026-09-28

**Authors:** Gi Yeon Lee, Jung Won Chun, Dong-eun Lee, Jun-Ha Jang, Woo Jin Lee, Hwang-Phill Kim, Seung-Tae Lee, Sang Myung Woo, Sun-Young Kong

**Affiliations:** 1 Targeted Therapy Branch, National Cancer Center, 323 Ilsan-ro, Ilsandong-gu, Goyang-si 10408, Republic of Korea; 2 Department of Laboratory Medicine, Yonsei University College of Medicine, 50-1 Yonsei-ro, Seodaemun-gu, Seoul 03722, Republic of Korea; 3 Center for Liver and Pancreatobiliary Cancer, National Cancer Center, 323 Ilsan-ro, Ilsandong-gu, Goyang-si 10408, Republic of Korea; 4 Biostatistics Collaboration Team, National Cancer Center, 323 Ilsan-ro, Ilsandong-gu, Goyang-si 10408, Republic of Korea; 5 Department of Public Health & AI, National Cancer Center Graduate School of Cancer Science and Policy, 323 Ilsan-ro, Ilsandong-gu, Goyang-si 10408, Republic of Korea; 6 IMBdx, 38 Teheran-ro 113-gil, Gangnam-gu, Seoul 06173, Republic of Korea; 7 Department of Laboratory Medicine, National Cancer Center, 323 Ilsan-ro, Ilsandong-gu, Goyang-si 10408, Republic of Korea; 8 Department of Cancer Biomedical Science, National Cancer Center Graduate School of Cancer Science and Policy, 323 Ilsan-ro, Ilsandong-gu, Goyang-si 10408, Republic of Korea

**Keywords:** circulating tumor DNA, pancreatic ductal adenocarcinoma, liquid biopsy, variant allele frequency, 5-fluorouracil, prognosis, longitudinal monitoring, CA19-9

## Abstract

Circulating tumor DNA (ctDNA) is a minimally invasive biomarker in pancreatic ductal adenocarcinoma (PDAC), but the prognostic information added by multigene ctDNA metrics beyond *KRAS* and carbohydrate antigen 19-9 (CA19-9) is uncertain. In this prospective observational pilot study, 51 patients with advanced PDAC receiving 5-fluorouracil-based chemotherapy underwent 118-gene ctDNA profiling at baseline and 4 and 8 weeks after treatment initiation. ctDNA burden was summarized with variant allele frequency (VAF) metrics: Sum VAF and Top4 VAF (*KRAS*, *TP53*, *CDKN2A*, *SMAD4*). Twelve-month progression-free survival (PFS) areas under the curve were 0.816 for Top4 VAF, 0.812 for Sum VAF, 0.788 for *KRAS* VAF, and 0.687 for CA19-9; the Top4–*KRAS* difference was not statistically significant (95% confidence interval, −0.120 to 0.131). Baseline ctDNA detection and high Top4 VAF remained associated with shorter PFS and overall survival after adjustment for clinical covariates. Detectable Top4 VAF at 4 weeks was associated with shorter PFS (hazard ratio, 5.739; *p* < 0.001). Top4 VAF changes differed across radiologic response categories at 8 but not 4 weeks; CA19-9 showed no significant paired change. These exploratory findings suggest that baseline and early on-treatment multigene ctDNA carries prognostic information in advanced PDAC, although superiority over *KRAS* or CA19-9 was not established.

## 1. Introduction

Pancreatic ductal adenocarcinoma (PDAC) remains one of the most lethal malignancies globally, characterized by late-stage diagnosis and exceptionally poor prognosis [1,2]. Despite advances in diagnostic techniques and chemotherapeutic treatments, the development of innovative biomarkers for monitoring treatment response, detecting early progression, and accurately predicting patient prognosis remains crucial.

Current PDAC monitoring relies primarily on imaging and serum carbohydrate antigen 19-9 (CA19-9), both of which have significant limitations. Imaging may miss subtle progression, particularly in the context of desmoplastic stroma, while CA19-9 is confounded by false positives in pancreatitis and false negatives in Lewis antigen-negative patients [2]. These limitations can contribute to delayed therapeutic decision-making and inadequate identification of treatment resistance.

Liquid biopsy via circulating tumor DNA (ctDNA) enables repeatable, minimally invasive monitoring of tumor burden, clonal dynamics, and treatment response [3,4]. Given the high prevalence of *KRAS* mutations in PDAC (~90%), extensive research has focused on detecting *KRAS* mutations within ctDNA [5,6]. However, single-gene approaches often overlook additional genetic alterations in patients without detectable *KRAS* mutations or those with low tumor burden, thereby diminishing their sensitivity and clinical utility [7].

Large-scale sequencing studies have identified canonical PDAC subtypes and broader mutational landscapes, supporting the rationale for multigene ctDNA profiling beyond *KRAS* alone [1,5,8]. Multigene approaches involving simultaneous analysis of clinically relevant mutations including *KRAS*, *TP53*, *SMAD4*, and *CDKN2A* may provide a broader representation of tumor-derived alterations and clonal dynamics [9]. The AlphaLiquid 100 minipanel (IMBdx, Seoul, Republic of Korea) is a next-generation sequencing (NGS)-based ctDNA platform analyzing 118 cancer-associated genes [10].

This study aimed to evaluate the prognostic value and longitudinal dynamics of the AlphaLiquid 100 ctDNA minipanel in patients with advanced PDAC receiving standard 5-fluorouracil (5-FU)-based chemotherapy. We hypothesized that comprehensive multigene ctDNA analyses would provide prognostic information beyond single-gene *KRAS* assays or conventional CA19-9.

## 2. Results

### 2.1. Patient Characteristics

A total of 51 patients with a median age of 64.0 years (range, 44–82 years) were included in this study (Table 1 and Figure 1). Of the 51 patients, 31 (60.8%) were males, and 20 (39.2%) were females. Most patients (82.4%, *n* = 42) were diagnosed with metastatic pancreatic cancer, 13.7% (*n* = 7) had locally advanced disease, and 3.9% (*n* = 2) had borderline resectable tumors. The tumor was located in the head of the pancreas in 49.0% (*n* = 25) of patients and in the body and tail in 51.0% (*n* = 26) of patients. Regarding treatment regimens, 66.7% (*n* = 34) of patients received FOLFIRINOX (5-FU, leucovorin, irinotecan, and oxaliplatin) as their 5-FU-based chemotherapy regimen, whereas 33.3% (*n* = 17) received liposomal irinotecan plus 5-FU/leucovorin. The 5-FU-based regimen was administered as first line in 64.7% (*n* = 33), second line in 31.4% (*n* = 16), and third line or beyond in 3.9% (*n* = 2) of patients. Most patients (76.5%, *n* = 39) had an Eastern Cooperative Oncology Group (ECOG) performance status of 0, whereas 23.5% (*n* = 12) had a status of 1. CA19-9 levels were elevated (>37 U/mL) in 72.5% (*n* = 37), normal (≤37 U/mL) in 25.5% (*n* = 13), and unknown in 2.0% (*n* = 1) of patients. Regarding radiologic outcomes, there was no statistically significant difference in treatment response between the baseline ctDNA-detected and not-detected groups. For the first response evaluated at initial follow-up, the rates of partial response (PR), stable disease (SD), and progressive disease (PD) were 23.3% (*n* = 10), 53.5% (*n* = 23), and 23.3% (*n* = 10) in the ctDNA-detected group, compared to 12.5% (*n* = 1), 62.5% (*n* = 5), and 25.0% (*n* = 2) in the not-detected group (*p* = 0.790). Similarly, the best overall response did not significantly differ between the two groups (*p* = 0.386), with a PR rate of 34.9% (*n* = 15) in patients with detectable baseline ctDNA versus 12.5% (*n* = 1) in those without.

### 2.2. Landscape of Somatic Mutations in ctDNA

The somatic mutational landscape of ctDNA revealed that KRAS and TP53 were the most frequently altered genes, recorded in 76% and 76% of the patients across the sampled time points, respectively (Figure 2). Other commonly mutated genes in the study cohort included *CDKN2A* (20%), *SMAD4* (18%), *ATM* (18%), and *EGFR* (18%). The mutational spectrum encompassed diverse alteration types, predominantly featuring missense mutations in key oncogenes and truncating mutations in tumor suppressor genes. Among *KRAS* alterations, codon 12 mutations predominated, with G12V, G12D, G12R, and G12C as the most common subtypes (Supplementary Figure S1).

### 2.3. Frequent Somatic Mutations and Domain Distribution in ctDNA

Mutation frequencies in plasma ctDNA from our National Cancer Center (NCC) cohort (*n* = 51) and in tumor tissue from The Cancer Genome Atlas (TCGA) (*n* = 185) showed similar overall patterns (Supplementary Figure S2). *KRAS* and *TP53* were the most frequently altered genes in both cohorts, detected in 76% and 76% of the NCC cohort and 74% and 59% of the TCGA cohort, respectively. *SMAD4* mutations were consistently observed in both datasets (18% and 21% in each cohort). Other commonly mutated genes included *CDKN2A*, *RNF43*, *GNAS* and *ARID1A*. Several genes identified in TCGA, including *TGFBR2*, *KMT2D* and *FAT1*, were not included in the ctDNA panel used in this study. Pathogenic and likely pathogenic variants were most frequently observed in *KRAS*, *TP53*, *CDKN2A*, *SMAD4*, and *ATM*. Figure 3 shows, as a descriptive illustration, the positional distribution of mutations along the encoded proteins for the ten genes altered in the largest number of patients, including *GNAS*. *KRAS* mutations predominantly clustered at codon 12 within the Ras domain. *TP53* alterations were broadly distributed across the DNA-binding and oligomerization domains. *SMAD4* mutations were clustered in the MH2 domain. *CDKN2A* mutations were mainly located in the ankyrin repeat regions.

### 2.4. Longitudinal Changes in ctDNA VAF During Early Chemotherapy

Changes in the plasma ctDNA mutation burden and variant allele frequencies (VAFs) are shown in Figure 4A–I for the 43 patients with baseline-detectable ctDNA. Patient-level distributions of mutation VAF across baseline, first follow-up (FU1), and second follow-up (FU2) are shown in Supplementary Figure S3, illustrating the overall downward shift in VAF after treatment initiation while preserving inter-patient heterogeneity. The total number of mutations detected per patient showed no statistically significant paired change (Figure 4A). However, significant reductions in multiple VAF-based quantitative metrics were observed after treatment. Sum VAF and Tier I/II VAF showed a marked decline from baseline to FU1 (Figure 4B,C). These reductions were maintained at FU2. Similarly, Mean VAF and Max VAF significantly decreased over time (Figure 4D,E). Gene-specific analysis revealed significant reductions in *KRAS* and *TP53* VAFs across time points (Figure 4F,G), whereas the summed VAF of variants in genes other than the Top4 genes showed no statistically significant paired change (Figure 4H). The Top4 VAF (*KRAS*, *TP53*, *CDKN2A*, *SMAD4*) also declined significantly (Figure 4I). At the patient level, longitudinal Top4 VAF trajectories demonstrated substantial heterogeneity by radiologic outcome (Supplementary Figure S4A,B). Patients achieving radiologic disease control generally showed reductions in Top4 VAF after treatment initiation, whereas those with progressive disease showed more heterogeneous patterns during follow-up. Among patients with baseline-detectable ctDNA (*n* = 43), the percentage change in Top4 VAF from baseline showed no statistically significant difference across Response Evaluation Criteria in Solid Tumors (RECIST) response categories at FU1 (Kruskal–Wallis *p* = 0.078), whereas a significant difference was observed at FU2 (*p* = 0.047) (Supplementary Figure S5A,B). Serum CA19-9 levels showed no statistically significant change from baseline to FU2 among the 47 patients measured at both time points (*p* = 0.299) (Figure 4J). CA19-9 was measured at FU1 in only 12 patients; in the 11 of these with a paired baseline measurement there was likewise no statistically significant change (*p* = 0.965). Because so few patients were measured at FU1, that time point is not shown in Figure 4J.

### 2.5. Prognostic Value of Baseline ctDNA VAF Metrics and CA19-9 in Pancreatic Cancer

During the follow-up period of 19.7 months (range, 2.4–25.6), the median progression-free survival (PFS) and overall survival (OS) were 5.4 months (95% confidence interval (CI), 4.2–7.3) and 9.3 months (95% CI, 8.3–18.5), respectively. Receiver operating characteristic (ROC) analysis showed that the areas under the curve (AUCs) for 12-month PFS were 0.816 for Top4 VAF, 0.812 for Sum VAF, 0.788 for KRAS VAF, and 0.687 for CA19-9 (Figure 5A). Although Top4 VAF showed a numerically higher AUC than KRAS VAF, the difference was not statistically significant in the paired bootstrap analysis (ΔAUC = 0.028; 95% CI, −0.120 to 0.131); no formal comparison with CA19-9 was performed.

Baseline CA19-9 (>37 U/mL) did not significantly stratify outcomes, showing no significant association with PFS (*p* = 0.114) or OS (*p* = 0.130) (Figure 5B,C). In contrast, ctDNA-based metrics demonstrated clear prognostic separation. Patients with elevated baseline Sum VAF (≥2.37%) had significantly shorter PFS (hazard ratio (HR) = 3.808; 95% CI: 1.797–8.069, *p* < 0.001) and OS (HR = 3.881; 95% CI: 1.791–8.410, *p* < 0.001) than those with low VAF (Figure 5D,E). Similarly, high Top4 VAF (≥0.57%) was associated with poorer PFS (HR = 2.796; 95% CI: 1.386–5.642, *p* = 0.003) and OS (HR = 3.207; 95% CI: 1.467–7.011, *p* = 0.002) (Figure 5F,G).

*KRAS* VAF alone showed a trend toward worse prognosis; however, the differences were not statistically significant (PFS: *p* = 0.155; OS: *p* = 0.077) (Figure 5H,I). These findings indicate that higher baseline Sum VAF and Top4 VAF were significantly associated with shorter survival outcomes, whereas baseline CA19-9 and single-gene *KRAS* VAF were not significantly associated with either outcome based on the survival curves.

In univariable and adjusted Cox proportional hazards models, baseline ctDNA detectability and Top4 VAF were significantly associated with both PFS and OS (Table 2). Specifically, baseline ctDNA detection remained associated with shorter PFS (adjusted HR, 5.435; *p* = 0.013) and OS (adjusted HR, 12.108; *p* = 0.002), while high Top4 VAF was also associated with shorter PFS (adjusted HR, 2.778; *p* = 0.014) and OS (adjusted HR, 2.944; *p* = 0.013). KRAS VAF was not significantly associated with either outcome after adjustment, whereas abnormal baseline CA19-9 was associated with PFS but not OS (Table 2).

### 2.6. Early On-Treatment ctDNA Persistence Is Associated with Inferior Survival Outcomes

The swimmer plot depicts longitudinal ctDNA detection status alongside disease progression and death during follow-up (Figure 6). Patients with detectable Top4 VAF at FU1 had significantly shorter PFS than those without detectable Top4 VAF (HR = 5.739, 95% CI, 2.618–12.580; *p* < 0.001) (Figure 7A), and OS was also significantly shorter in these patients (HR = 6.008, 95% CI, 2.574–14.030; *p* < 0.001) (Figure 7B). Detectable *KRAS* VAF at FU1 was also associated with significantly shorter PFS (HR = 4.389, 95% CI, 2.067–9.319; *p* < 0.001) (Figure 7C) and OS (HR = 4.718; 95% CI, 2.215–10.05; *p* < 0.001) (Figure 7D). This prognostic association persisted at FU2: detectable Top4 VAF was associated with shorter PFS (HR = 2.459; 95% CI, 1.268–4.770; *p* = 0.006) (Figure 7E) and OS (HR = 2.201; 95% CI, 1.032–4.690; *p* = 0.036) (Figure 7F), and detectable *KRAS* VAF was similarly associated with worse PFS (HR = 2.724; 95% CI, 1.415–5.244; *p* = 0.002) (Figure 7G) and OS (HR = 2.973; 95% CI, 1.423–6.213; *p* = 0.002) (Figure 7H).

### 2.7. Longitudinal ctDNA Detection Transitions and Patient-Level Variant Dynamics

Supplementary Figure S6A shows longitudinal changes in ctDNA detectability across the three sampling time points. Among 51 patients, ctDNA was detected in 43 patients at baseline and in 44 patients at FU1, whereas the number of ctDNA-detected patients decreased to 38 at FU2, indicating an overall shift toward reduced ctDNA detectability during treatment. Notably, a subset of patients showed conversion between detected and not detected states across follow-up, reflecting inter-individual heterogeneity in early molecular response patterns. The two patient-level examples showed contrasting longitudinal variant-detection patterns (Supplementary Figure S6B,C). In the patient with an unfavorable clinical course (S6B; C288-023), multiple alterations remained persistently detectable across baseline, FU1, and FU2, with limited clearance and dynamic persistence of ctDNA signals during treatment. In contrast, the patient with a favorable clinical course (S6C; C288-012) showed an early contraction of detectable alterations during follow-up, characterized by transient loss of detectability of key alterations at FU1 and overall attenuation of ctDNA signals.

## 3. Discussion

This study evaluated a 118-gene ctDNA panel (AlphaLiquid 100) for noninvasive longitudinal monitoring and prognostication in patients with advanced PDAC receiving 5-FU-based chemotherapy. The prognostic associations of multigene VAF metrics support their investigation as complementary markers of tumor burden [11], but do not establish superiority over *KRAS* VAF or CA19-9. Consistent with our findings, Shibuki et al. (2025) reported prospective results from the ARTEMIS-PC study showing numerically higher AUCs for ctDNA VAF than for CA19-9 in predicting disease control in unresectable PDAC; that report is available in abstract form and used a patient-specific approach [12]. The descriptive cross-cohort comparison with TCGA tumor tissue is consistent with ctDNA reflecting tumor-derived alterations, although the two datasets differ in specimen type and variant filtering. Protein domain-level mapping was a separate descriptive analysis and does not establish functional or clinical validity.

Importantly, our findings support further evaluation of longitudinal ctDNA kinetics as an early on-treatment biomarker. Longitudinal VAF metrics may reflect changes in molecular burden that are not captured by counting detected variants alone. These findings suggest that longitudinal ctDNA kinetics may provide complementary information during treatment; however, their comparative value relative to conventional imaging or longitudinal CA19-9 monitoring requires further investigation. In addition, Edland et al. demonstrated that serial *KRAS* ctDNA monitoring in advanced PDAC predicted clinical outcomes and detected disease progression a median of 23 days earlier than radiological imaging, supporting the concept that molecular dynamics may precede radiologic change [13]. While their analysis focused on *KRAS*-only tracking, our multigene minipanel-based approach extends this concept by enabling broader and scalable longitudinal monitoring beyond a single alteration. Similarly, Lapin et al. reported that comprehensive longitudinal ctDNA measurements using targeted NGS improved prediction of clinical outcomes and enabled dynamic tracking of disease progression in advanced pancreatic cancer, supporting the clinical relevance of VAF-based summary metrics for monitoring [14]. Prior studies similarly support investigating persistent ctDNA detection as a prognostic marker during treatment [15,16,17].

These findings have several clinical implications. First, early identification of patients without a molecular response might inform timely therapeutic adjustment. Second, whether ctDNA can guide treatment de-escalation or imaging schedules requires prospective interventional evaluation. Third, integrating ctDNA-based molecular monitoring into clinical trial designs may allow adaptive stratification based on early molecular response rather than conventional static criteria such as RECIST or CA19-9 [6,18]. In this regard, Steiniche et al. (2025) proposed a ctDNA-RECIST framework that applies RECIST-like categories to early ctDNA kinetics in metastatic PDAC and demonstrated the prognostic impact of early molecular response [19]. Our VAF-based summary measures (e.g., Top4 VAF and Sum VAF) may provide practical candidate metrics to parameterize or refine such molecular response criteria, warranting prospective validation alongside radiologic response assessment. Collectively, comprehensive ctDNA profiling using a multigene panel may support patient stratification and earlier detection of treatment resistance, and liquid biopsy-based longitudinal monitoring holds promise for more personalized treatment strategies for patients with this aggressive malignancy, provided that these applications are validated prospectively.

Perioperatively, tumor-informed assays indicate that ctDNA positivity in the molecular residual disease (MRD) window (2–12 weeks after resection) and during surveillance stratifies disease-free survival, and genotype-specific differences (e.g., *KRAS* G12V) have been noted. For example, Botta et al. (2024) reported that perioperative tumor-informed ctDNA positivity and ctDNA detection during surveillance were strongly associated with inferior survival outcomes in pancreatic adenocarcinoma, supporting ctDNA as a clinically relevant MRD biomarker [20]. While this perioperative MRD context differs from our treatment-monitoring setting in advanced PDAC, both lines of evidence highlight the prognostic value of ctDNA detectability. In advanced PDAC, prospective studies show that longitudinal ctDNA dynamics measured using targeted next-generation sequencing panels or *KRAS*-based assays track chemotherapy response and disease progression, and baseline ctDNA detectability is associated with inferior PFS [14,21,22,23,24]. Our results are consistent with Wei et al. (2019), who demonstrated that serial cell-free DNA (cfDNA) profiling using a multigene NGS approach during FOLFIRINOX reflected changes in tumor burden and treatment response in pancreatic cancer [25]. Compared with that study, our cohort is larger and includes patients treated with liposomal irinotecan plus 5-FU/leucovorin as well as FOLFIRINOX, further supporting the practical utility of multigene VAF-derived summary metrics for longitudinal monitoring. Beyond targeted panels, ctDNA whole-exome sequencing has been shown to delineate organ-specific metastatic tropism and to surface actionable alterations in rapidly progressing PDAC, with potential relevance to treatment escalation or trial enrolment [26]. The role of ctDNA in guiding treatment decisions in PDAC nevertheless remains under evaluation [27]. Fragmentomics, which analyzes genome-wide fragmentation patterns, size selection, and nucleosome footprints, may offer additional sensitivity and biological interpretability, although these approaches have been evaluated largely outside PDAC. Broader liquid biopsy frameworks on MRD detection, unique molecular identifier (UMI)/error modeling, and cross-platform concordance contextualize these findings [28,29,30,31].

This study has several limitations. The cohort size was small, and matched tumor tissue sequencing data were not available for direct comparison. The limited sample size also resulted in uncertainty in comparisons of discriminatory performance between biomarkers; therefore, the observed numerical differences in AUCs should be interpreted as exploratory. The VAF cutoffs used for survival stratification were derived from the same dataset by time-dependent ROC analysis and are therefore at risk of optimism, and longitudinal CA19-9 measurements were available for only 12 patients at FU1, which limits the comparison with ctDNA dynamics at that time point. Furthermore, our cohort was limited to patients receiving 5-FU-based chemotherapy, and those treated with gemcitabine-based regimens were not included. Consequently, the utility and dynamic patterns of the ctDNA panel across different chemotherapeutic agents remain to be fully elucidated. Future comparative studies involving more diverse treatment cohorts are warranted to validate the cross-regimen efficacy of ctDNA-based monitoring. Although the AlphaLiquid 100 panel includes a wide array of clinically relevant genes, it does not fully capture the mutational breadth observed in whole-exome or genome-level datasets. Furthermore, the lack of multiomics integration, including exosomal RNA, protein biomarkers, and circulating tumor cells, limits the scope of biological interpretation. Nonetheless, our findings underscore the potential of ctDNA not only as a molecular proxy for tumor burden but also as a potential tool for longitudinal assessment during treatment in PDAC. Further large-scale prospective studies are needed to validate these findings and establish ctDNA-guided algorithms for the routine management of PDAC [32].

## 4. Materials and Methods

### 4.1. Patient Recruitment and Collection of Clinical Data

This prospective observational pilot study included patients aged ≥18 years with pathologically confirmed PDAC who received 5-FU-based chemotherapy at the National Cancer Center, Korea, between November 2021 and February 2024.

Clinical data were systematically collected, including patient demographics, cancer stage, tumor location, Eastern Cooperative Oncology Group (ECOG) performance status, treatment regimen, and serum tumor markers including CA19-9, which was recorded at baseline and at the FU1 and FU2 blood sampling visits when available; results reported as <5 U/mL were retained as <5 U/mL. Tumor response was radiographically assessed using computed tomography or magnetic resonance imaging approximately every two months according to Response Evaluation Criteria in Solid Tumors (RECIST) version 1.1 [18]. Progression-free survival (PFS) was defined as time from chemotherapy initiation to disease progression or death, and overall survival (OS) as time from chemotherapy initiation to death from any cause.

### 4.2. Blood Sample Collection and cfDNA Extraction

Blood samples were collected at three time points: baseline (pre-treatment), first follow-up (FU1; ~4 weeks after treatment initiation), and second follow-up (FU2; ~8 weeks after treatment initiation). Eight to ten milliliters of whole blood was collected in Streck tubes. Samples were centrifuged at 1500× *g* for 15 min, followed by additional centrifugation at 16,000× *g* for 10 min to eliminate cell debris. Plasma was isolated within 20 min of blood collection and stored at −80 °C until extraction [33].

Cell-free DNA was extracted from 2–4 mL of plasma using the Maxwell^®^ RSC cfDNA Plasma Kit (Promega, Madison, WI, USA) according to manufacturer’s instructions and quantified using a 4200 TapeStation (Agilent Technologies, Santa Clara, CA, USA). For matched germline reference, genomic DNA was extracted from buffy coat using the Maxwell^®^ RSC Blood DNA Kit and sequenced in parallel to filter germline variants and clonal hematopoiesis of indeterminate potential (CHIP).

### 4.3. Targeted Deep Sequencing and Variant Identification

NGS libraries were constructed using the IMBdx NGS DNA Library Prep Kit (IMBdx, Seoul, Republic of Korea). Solution-based target enrichment was performed using the AlphaLiquid 100 target capture panel comprising 118 cancer-related genes (Supplementary Table S1). Libraries were sequenced on the Illumina NovaSeq 6000 platform (Illumina, San Diego, CA, USA) in 2 × 150 bp paired-end mode [10,34].

Somatic variants including single-nucleotide variants (SNVs), small insertions/deletions (INDELs), copy number alterations, and gene fusions were identified using the AlphaLiquid 100 analysis pipeline [10]. Variants present in matched leukocyte DNA were removed to exclude germline variants and CHIP. Patients were classified as “Detected” or “Not Detected” based on presence of ≥1 somatic variant with variant allele frequency (VAF) ≥ 0.1% at baseline. Only variants with VAF ≥ 0.1% were counted at each time point. Sum VAF was defined as the sum of the VAFs of all detected variants; Mean VAF and Max VAF as the mean and maximum variant VAF per sample; Tier I/II VAF as the summed VAF of variants assigned to clinical significance Tier I or II; *KRAS* VAF and *TP53* VAF as the summed VAF of variants in the respective gene; Top4 VAF as the sum of VAFs for *KRAS*, *TP53*, *SMAD4*, and *CDKN2A*; and Other-gene VAF as the summed VAF of variants in the remaining genes. On-treatment ctDNA kinetics were assessed by comparing VAF metrics between follow-up time points and baseline in patients with baseline-detectable ctDNA, and molecular response was summarized as the percentage change in Top4 VAF from baseline. For the early on-treatment analyses, patients were stratified at each follow-up time point according to detectability of Top4 VAF or *KRAS* VAF (VAF ≥ 0.1%). Mutation frequencies were compared descriptively with the TCGA PDAC tumor tissue dataset (*n* = 185), and variant positions were mapped onto the encoded protein for the ten genes altered in the largest number of patients, ranked by the number of patients carrying at least one variant in the gene. Each gene was drawn on the protein of a single reference transcript; records reported on a second isoform whose residue numbering does not correspond to that protein, and records without a protein position, were not plotted. Protein regions were taken from public domain annotations.

### 4.4. Statistical Analysis

Categorical variables were summarized as frequencies and proportions, and continuous variables as medians with ranges or interquartile ranges, as appropriate. Time-dependent receiver operating characteristic (ROC) analysis was performed to evaluate the discriminatory performance of baseline biomarkers for 12-month progression-free survival (PFS) and to determine VAF cutoffs for survival stratification; because these cutoffs were derived from the same dataset, they are exploratory. The difference in 12-month AUC between Top4 VAF and *KRAS* VAF was assessed with a paired bootstrap procedure (5000 resamples); no formal comparison with CA19-9 was performed. Longitudinal changes in VAF metrics and in serum CA19-9 between time points were compared with the Wilcoxon signed-rank test for paired samples using available pairs without imputation, and differences in the percentage change in Top4 VAF across RECIST response categories were assessed with the Kruskal–Wallis test. Baseline characteristics were compared with the Fisher exact or chi-square test and the Mann–Whitney U test. Survival outcomes were analyzed using the Kaplan–Meier method, log-rank test, and Cox proportional hazards regression models. Univariable Cox proportional hazards regression models were used to evaluate associations between individual biomarkers and survival outcomes. Each biomarker was subsequently evaluated in a separate multivariable Cox proportional hazards model adjusted for disease stage, Eastern Cooperative Oncology Group (ECOG) performance status, and chemotherapy regimen. These covariates were selected based on their clinical relevance. Statistical analyses were performed using R version 4.4.2 (R Foundation for Statistical Computing, Vienna, Austria); the baseline CA19-9 summary in Table 1 was re-aggregated for this revision in Python 3.12 (pandas, NumPy). A two-sided *p* value of <0.05 was considered statistically significant, and *p* values are reported to three decimal places (*p* < 0.001 where applicable). As this was a prospective observational pilot study, no formal sample size or power calculation was performed. Instead, the sample size was determined by feasibility, based on the number of consecutively eligible patients enrolled during the predefined study period, and was intended to provide preliminary effect-size estimates for future validation studies.

## 5. Conclusions

This prospective pilot study suggests that multigene ctDNA profiling using a 118-gene targeted panel may provide prognostic information in advanced PDAC treated with 5-FU-based chemotherapy. Baseline ctDNA burden and early on-treatment ctDNA detection were associated with survival outcomes, while longitudinal changes in ctDNA VAF may provide complementary information for monitoring molecular changes during treatment. Future large-scale prospective studies are warranted to validate these findings and establish standardized thresholds for clinical implementation.

## Figures and Tables

**Figure 1:**
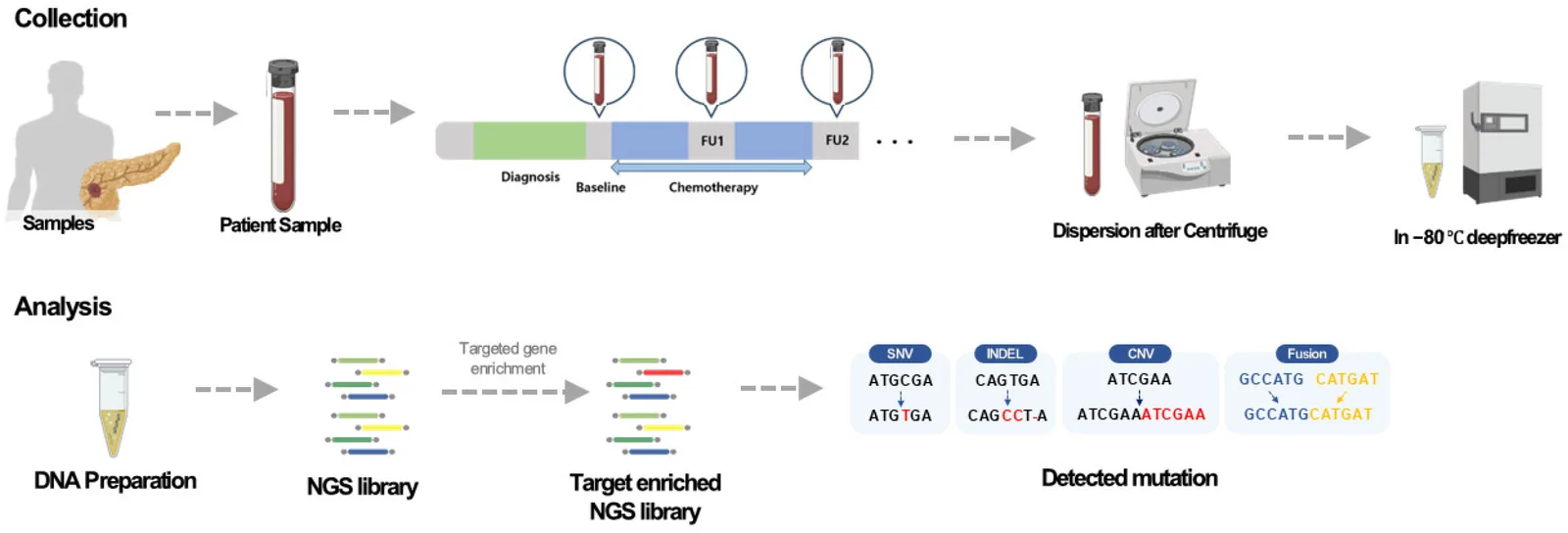
Study workflow. Peripheral blood samples were collected from patients with advanced PDAC at three time points: baseline (pre-treatment), first follow-up (FU1, approximately 4 weeks), and second follow-up (FU2, approximately 8 weeks) after initiation of 5-FU-based chemotherapy. Plasma was isolated by a two-step centrifugation protocol and stored at −80 °C. Cell-free DNA (cfDNA) was extracted and subjected to targeted NGS using the AlphaLiquid 100 118-gene panel; detected alterations comprised single-nucleotide variants (SNVs), small insertions/deletions (INDELs), copy number variations (CNVs), and gene fusions. Created in BioRender. Kong, S. (2026) https://BioRender.com/rc6r85x.

**Figure 2:**
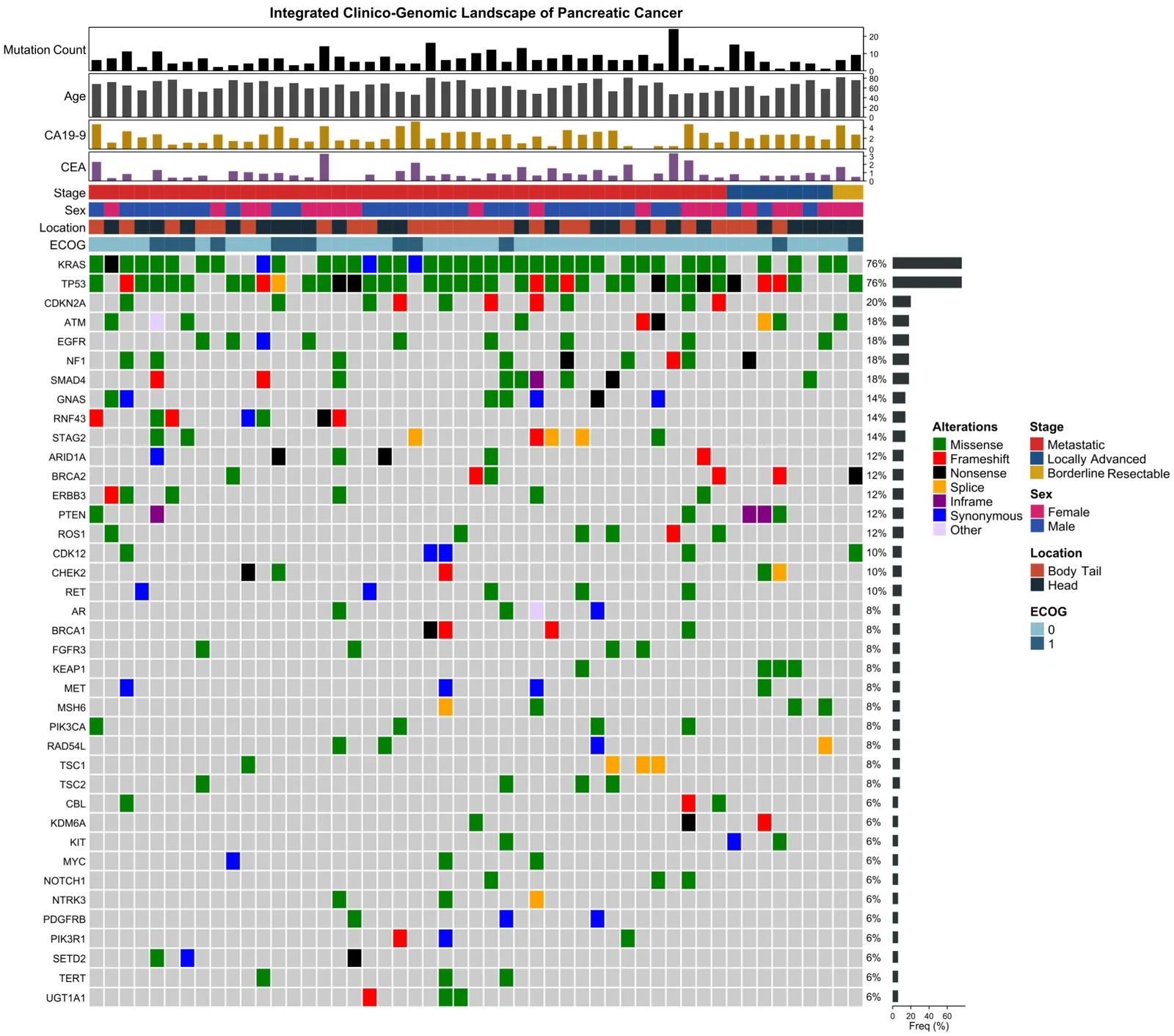
Oncoprint of somatic alterations detected in plasma ctDNA with the 118-gene panel. Each column represents an individual patient (*n* = 51) and each row a gene; colored tiles indicate the alteration type as defined in the key. The top bar plot shows the per-patient number of variant records, and the annotation tracks show age, serum CA19-9 and carcinoembryonic antigen (CEA) after log transformation, clinical stage, sex, tumor location, and ECOG performance status. Gene frequencies refer to the patient-level catalogue of variant records across the study time points.

**Figure 3:**
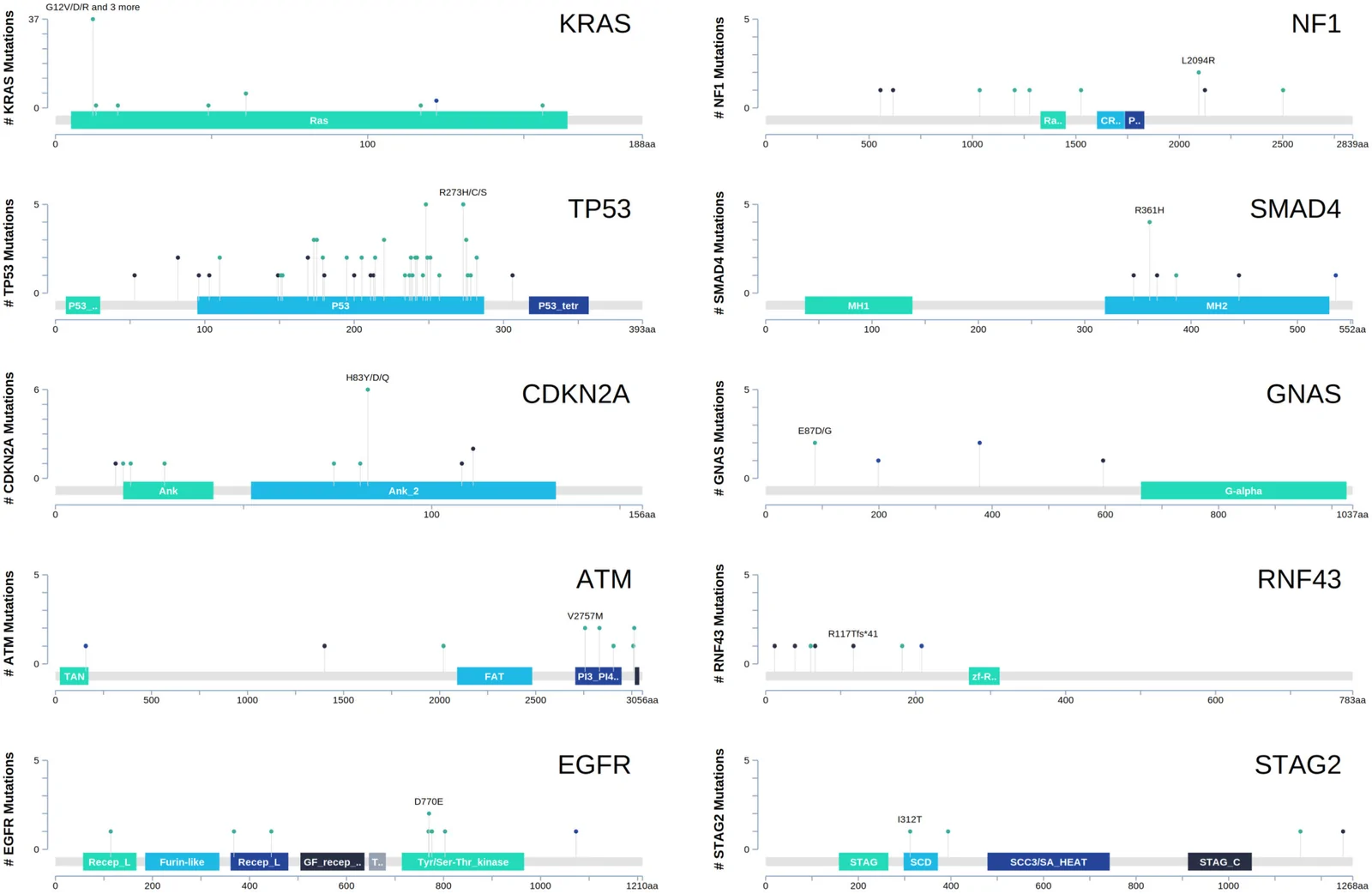
Positions of plasma ctDNA variants along the encoded protein for the ten genes altered in the largest number of patients, ordered by patient frequency. In each panel the grey bar is the protein from residue 1 to its full length, coloured boxes depict protein regions, and the y axis is the number of variant records at each residue; one representative residue is labelled. Dot colour indicates the reported consequence class (missense; truncating, comprising nonsense and frameshift changes; other classes). A residue carrying more than one class is drawn as a single dot in the colour of its most frequent class; so, colours do not enumerate every record. One protein is shown per gene, including *CDKN2A* p16INK4a, *EGFR* canonical isoform and *GNAS* XLαs. Six records without protein positions and three reported on a second isoform whose numbering does not correspond to the protein shown are not drawn. These omissions apply only to this figure; the source dataset and statistical analyses were unchanged, and gene frequencies were calculated from the full variant catalogue.

**Figure 4:**
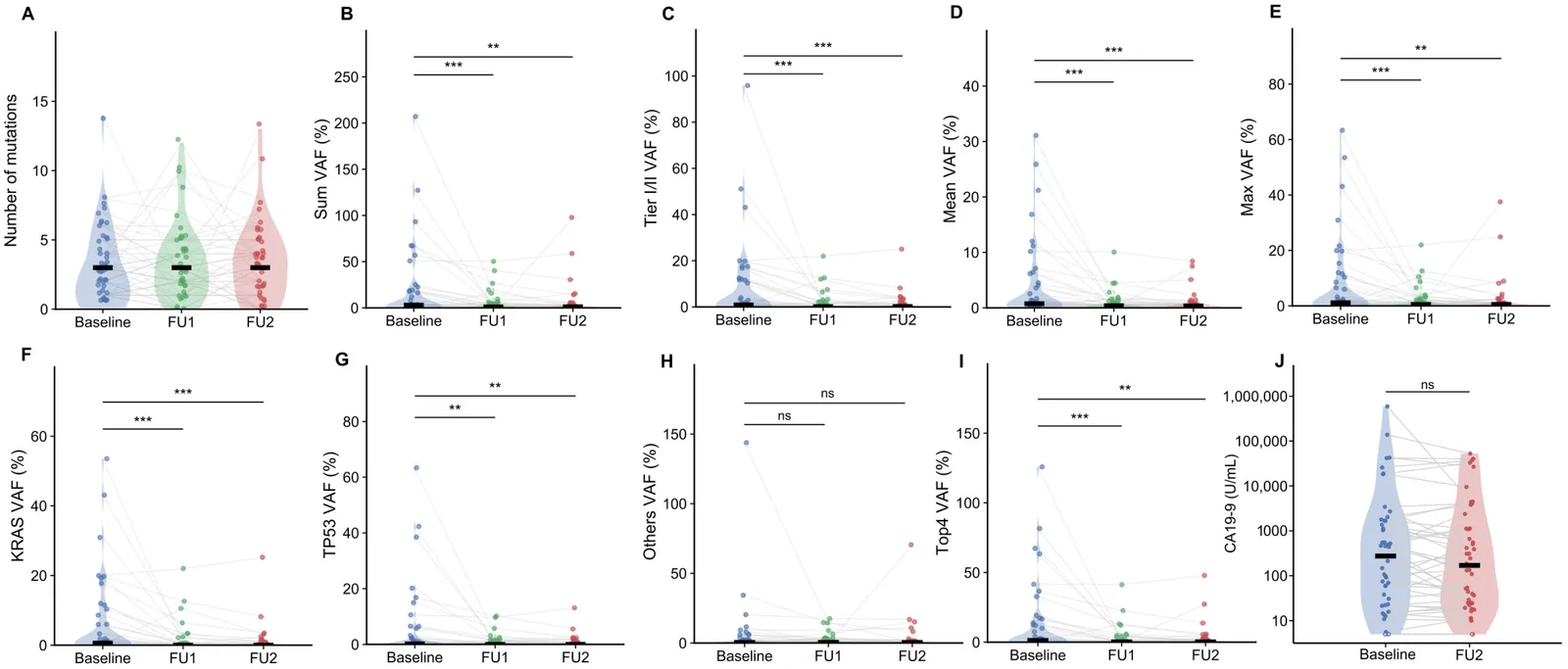
Longitudinal changes in ctDNA VAF-based metrics and serum CA19-9 during 5-FU-based chemotherapy (baseline, FU1, FU2; patients with baseline-detectable ctDNA, *n* = 43, for panels (**A**–**I**)). (**A**) Number of detected mutations per patient. (**B**) Sum VAF. (**C**) Tier I/II VAF. (**D**) Mean VAF. (**E**) Max VAF. (**F**) *KRAS* VAF. (**G**) *TP53* VAF. (**H**) Other-gene VAF, defined as the summed VAF of variants in genes other than *KRAS*, *TP53*, *CDKN2A*, and *SMAD4* (labelled “Others VAF” in the panel). (**I**) Top4 VAF (*KRAS*, *TP53*, *CDKN2A*, and *SMAD4*). (**J**) Serum CA19-9 (U/mL) at baseline and FU2 in the 47 patients measured at both time points, shown on a log10-scaled axis labelled in original units; black bars indicate medians in original units (274 and 170 U/mL), and values reported as <5 U/mL are plotted at 5 U/mL as open circles. CA19-9 was measured at FU1 in 12 patients and that time point is not shown. In all panels, violins show the distribution, points represent individual patients, grey lines connect measurements from the same patient, and black bars indicate medians. Comparisons between time points were performed with the Wilcoxon signed-rank test for paired samples on original-scale values (CA19-9: paired *n* = 47 for baseline vs. FU2; the log10 axis in panel (**J**) is for display only). ** *p* < 0.01, *** *p* < 0.001; ns, not significant.

**Figure 5:**
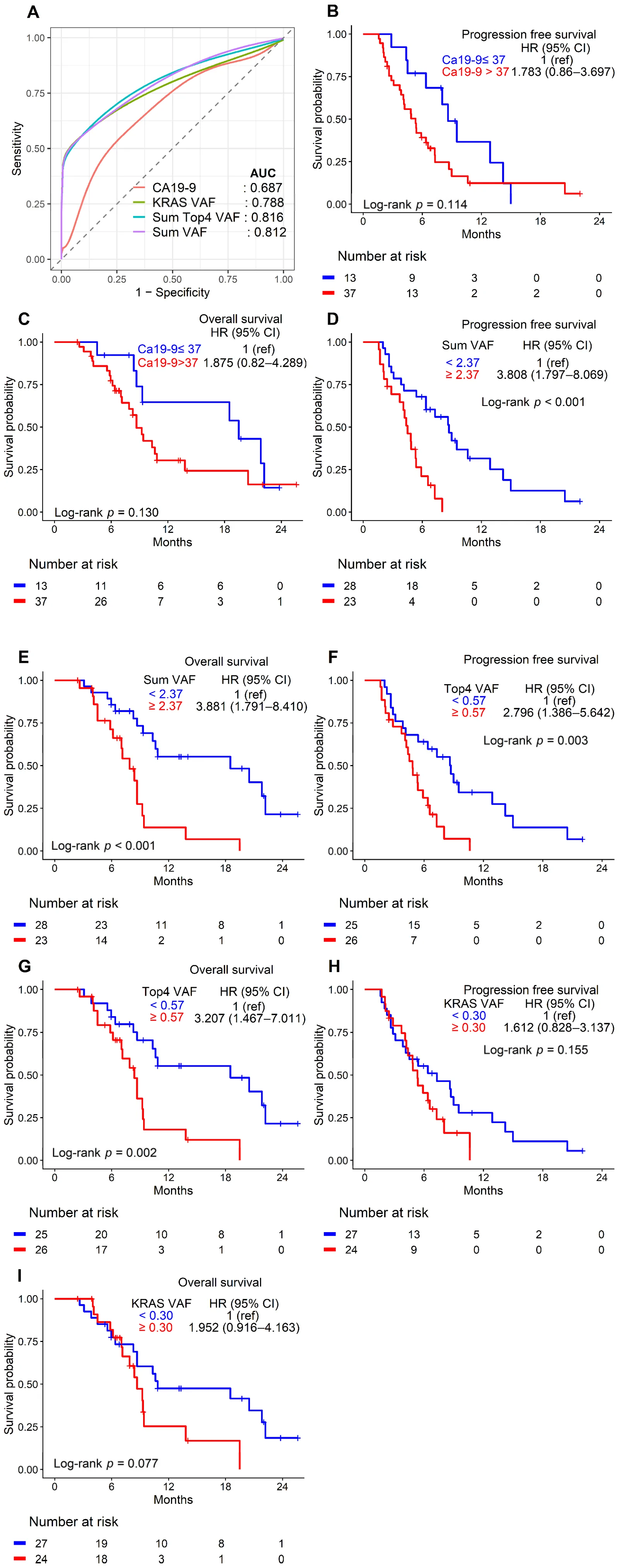
Prognostic performance of ctDNA VAF-based metrics and CA19-9 for survival outcomes in pancreatic cancer. (**A**) Time-dependent ROC curves showing the discriminatory power of baseline biomarkers for predicting 12-month PFS. Top4 VAF (AUC = 0.816) and Sum VAF (AUC = 0.812) showed numerically higher AUCs than KRAS VAF (AUC = 0.788) and serum CA19-9 (AUC = 0.687); the Top4 VAF–*KRAS* VAF difference was not statistically significant. (**B**,**C**) Kaplan–Meier analysis by CA19-9 levels (cutoff = 37 U/mL) showed no significant differences in progression-free survival (*p* = 0.114) or overall survival (*p* = 0.130). (**D**,**E**) Patients with high Sum VAF (≥2.37%) had significantly shorter progression-free (*p* < 0.001) and overall survival (*p* < 0.001). (**F**,**G**) Similarly, high Top4 VAF (≥0.57%) was associated with worse progression-free (*p* = 0.003) and overall survival (*p* = 0.002). (**H**,**I**) While *KRAS* VAF (cutoff = 0.30%) showed a trend toward poorer outcomes, the differences were not statistically significant.

**Figure 6:**
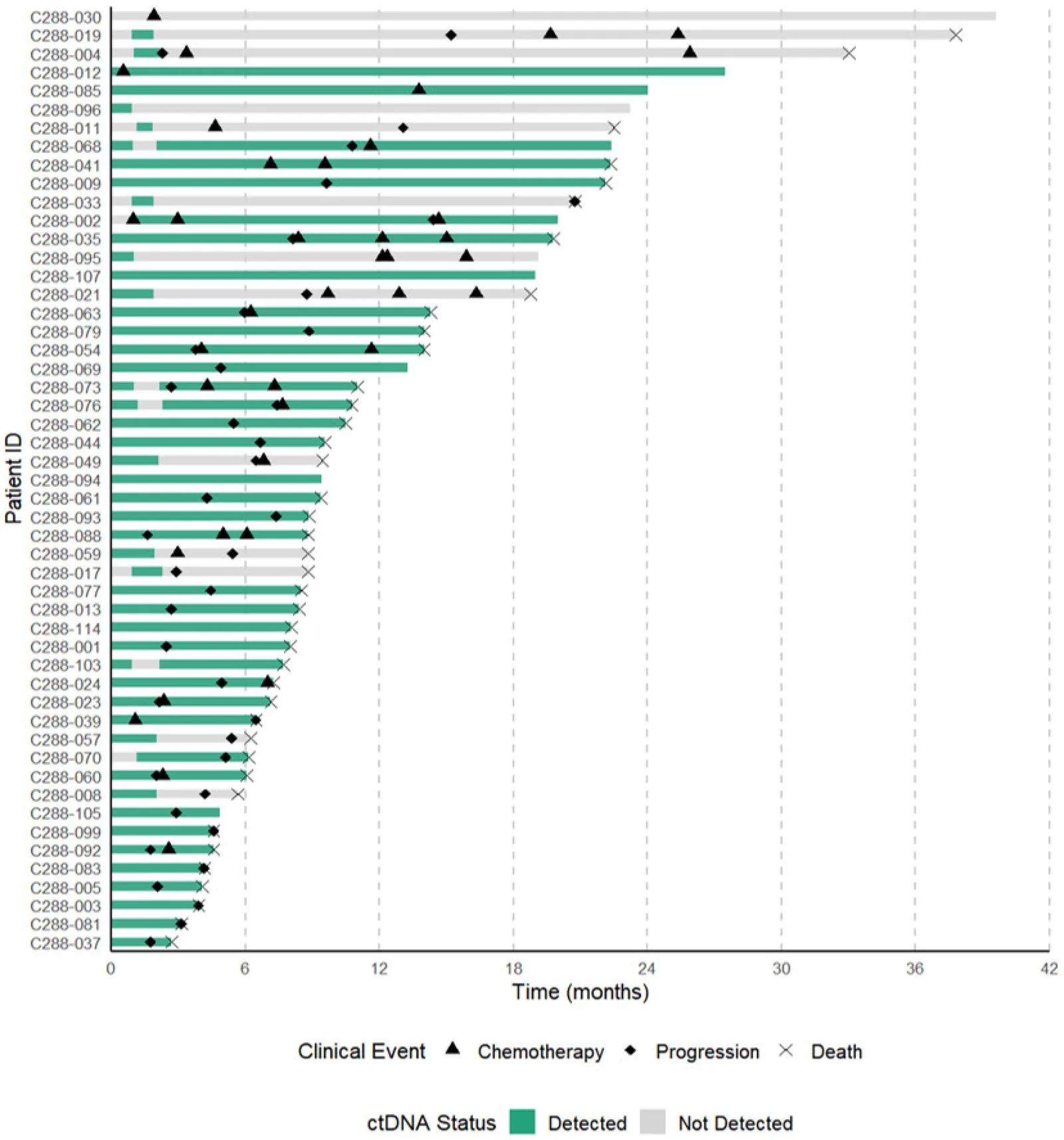
Swimmer plot of the clinical course of the 51 patients. Each horizontal bar represents one patient from initiation of 5-FU-based chemotherapy to progression, death, or last follow-up (months). Bar segments are colored by ctDNA detection status (green, detected; grey, not detected). Symbols mark clinical events: triangles, chemotherapy initiation; diamonds, disease progression; crosses, death.

**Figure 7:**
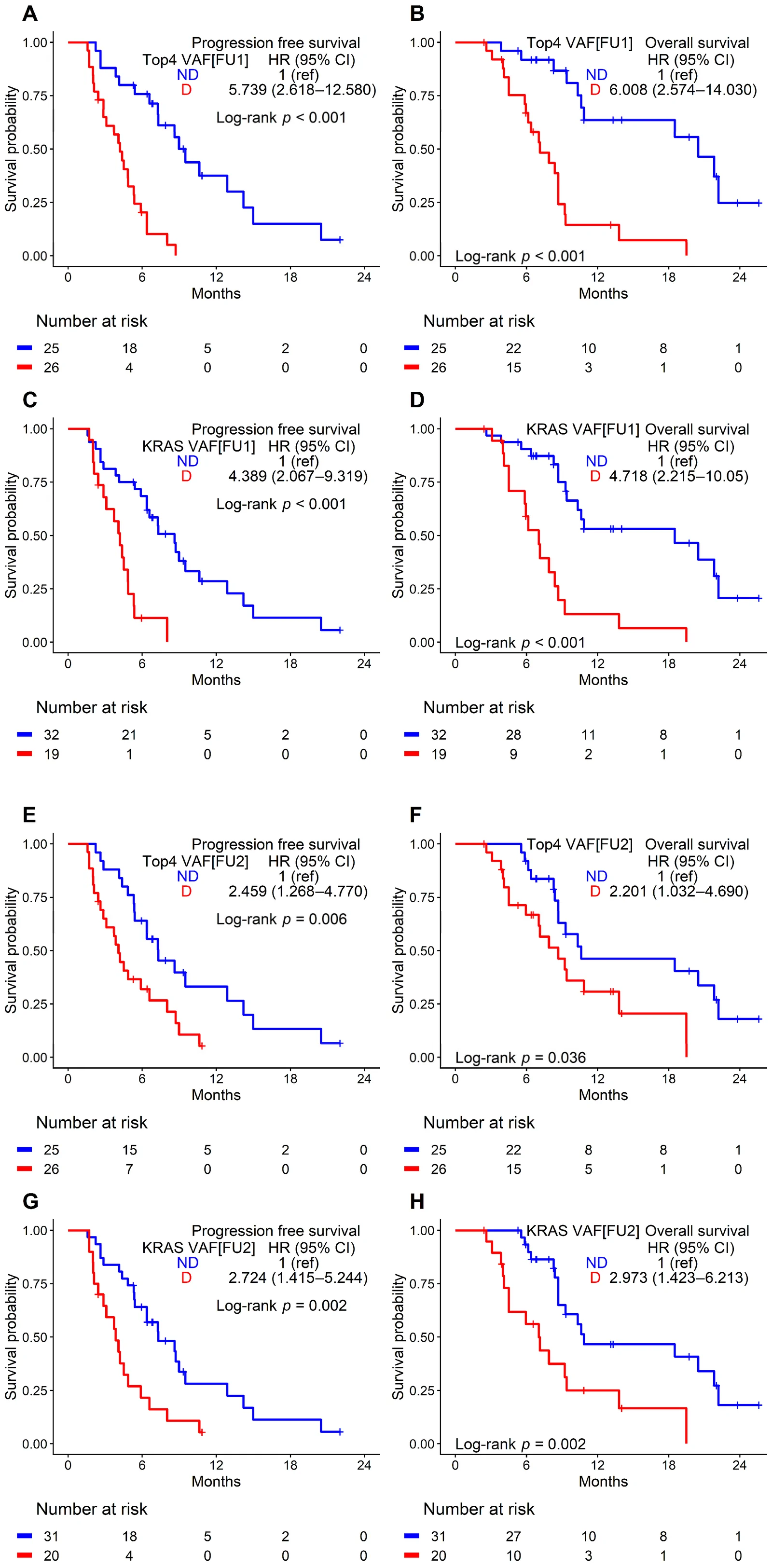
Prognostic significance of early on-treatment ctDNA detection during 5-FU-based chemotherapy. (**A**–**H**) Kaplan–Meier analyses evaluating the prognostic impact of early on-treatment ctDNA detection status. Patients were stratified by ctDNA detection status at each follow-up time point: “Detected (D)” denotes patients in whom Top4 VAF or *KRAS* VAF was detectable (VAF ≥ 0.1%), and “Not Detected (ND)” denotes patients with undetectable ctDNA. (**A**) PFS by Top4 VAF detection status at FU1. (**B**) OS by Top4 VAF detection status at FU1. (**C**) PFS by *KRAS* VAF detection status at FU1. (**D**) OS by *KRAS* VAF detection status at FU1. (**E**) PFS by Top4 VAF detection status at FU2. (**F**) OS by Top4 VAF detection status at FU2. (**G**) PFS by *KRAS* VAF detection status at FU2. (**H**) OS by *KRAS* VAF detection status at FU2.

**Table 1:** Baseline clinicopathological characteristics of patients with pancreatic ductal adenocarcinoma stratified by ctDNA detection status.

Characteristic	Total (*n* = 51)	Detected (*n* = 43)	Not Detected (*n* = 8)	*p* Value
Age, years, median (range)	64 (44–82)	65 (44–82)	61 (54–76)	0.795
Sex				0.696
Male	31 (60.8)	27 (62.8)	4 (50.0)	
Female	20 (39.2)	16 (37.2)	4 (50.0)	
Clinical stage				0.518
Metastatic	42 (82.4)	36 (83.7)	6 (75.0)	
Locally advanced	7 (13.7)	5 (11.6)	2 (25.0)	
Borderline resectable	2 (3.9)	2 (4.7)	0 (0.0)	
ECOG PS				0.076
0	39 (76.5)	35 (81.4)	4 (50.0)	
1	12 (23.5)	8 (18.6)	4 (50.0)	
Tumor location				0.140
Head	25 (49.0)	19 (44.2)	6 (75.0)	
Body/Tail	26 (51.0)	24 (55.8)	2 (25.0)	
5-FU-based regimen				>0.999
FOLFIRINOX	34 (66.7)	29 (67.4)	5 (62.5)	
5-FU + nanoliposomal irinotecan	17 (33.3)	14 (32.6)	3 (37.5)	
Line of therapy				0.365
First line	33 (64.7)	28 (65.1)	5 (62.5)	
Second line	16 (31.4)	14 (32.6)	2 (25.0)	
≥Third line	2 (3.9)	1 (2.3)	1 (12.5)	
CA19-9, U/mL, median (IQR)	244.0 (32.6–1585.0)	482.0 (61.9–1756.3)	45.4 (15.5–100.6)	0.027
CA19-9 level				
Abnormal (>37 U/mL)	37 (72.5)	33 (76.7)	4 (50.0)	0.309
Normal (≤37 U/mL)	13 (25.5)	9 (20.9)	4 (50.0)	
Unknown	1 (2.0)	1 (2.3)	0 (0.0)	
CEA, ng/mL, median (IQR)	5.3 (2.1–14.9)	5.9 (2.8–15.0)	3.0 (1.5–7.4)	0.016
First response				0.790
PR	11 (21.6)	10 (23.3)	1 (12.5)	
SD	28 (54.9)	23 (53.5)	5 (62.5)	
PD	12 (23.5)	10 (23.3)	2 (25.0)	
Best response				0.386
PR	16 (31.4)	15 (34.9)	1 (12.5)	
SD	22 (43.1)	17 (39.5)	5 (62.5)	
PD	13 (25.5)	11 (25.6)	2 (25.0)	

NOTE: Data are presented as *n* (%) unless otherwise specified. ctDNA detection was defined as the presence of ≥1 somatic variant with VAF ≥ 0.1% at baseline. Abbreviations: ctDNA, circulating tumor DNA; ECOG PS, Eastern Cooperative Oncology Group Performance Status; 5-FU, 5-fluorouracil; CA19-9, carbohydrate antigen 19-9; CEA, carcinoembryonic antigen; PR, partial response; SD, stable disease; PD, progressive disease; IQR, interquartile range; VAF, variant allele frequency. *p* values were calculated using Fisher exact test or chi-square test for categorical variables, and Mann–Whitney *U* test for continuous variables. Baseline CA19-9 was available for 50 patients (detected, *n* = 42; not detected, *n* = 8); the categorical comparison included the unknown category as a third level, and three values reported as <5 U/mL were treated as tied lowest ranks.

**Table 2:** Univariable and Adjusted Cox proportional hazards regression models for progression-free survival and overall survival.

Variable	Progression-Free Survival				Overall Survival			
	Univariable		Adjusted *		Univariable		Adjusted *	
	HR (95% CI)	*p*	HR (95% CI)	*p*	HR (95% CI)	*p*	HR (95% CI)	*p*
Full cohort (*n* = 51)								
ctDNA detection (detected vs. not detected)	4.791 (1.410–16.286)	**0.012**	5.435 (1.439–20.535)	**0.013**	10.622 (2.361–47.780)	**0.002**	12.108 (2.508–58.450)	**0.002**
Top4 VAF (high vs. low)	2.796 (1.386–5.642)	**0.004**	2.778 (1.235–6.250)	**0.014**	3.207 (1.467–7.011)	**0.004**	2.944 (1.257–6.899)	**0.013**
KRAS VAF (high vs. low)	1.612 (0.828–3.137)	0.160	1.308 (0.623–2.744)	0.478	1.952 (0.9156–4.163)	0.083	1.614 (0.716–3.639)	0.249
CA 19-9 (abnormal vs. normal)	1.783 (0.860–3.697)	0.120	2.227 (1.027–4.832)	**0.043**	1.875 (0.820–4.289)	0.136	2.108 (0.910–4.883)	0.082

NOTE: Top4 VAF = sum of VAF for KRAS, TP53, CDKN2A, and SMAD4. High vs. low was determined by time-dependent ROC analysis for 12-month PFS prediction (Top4 VAF cutoff: 0.57%). KRAS VAF cutoff: ≥0.30%. Patients with undetectable ctDNA were classified as VAF = 0 (low group). * Each biomarker was evaluated in a separate multivariable Cox proportional hazards model adjusted for disease stage, Eastern Cooperative Oncology Group (ECOG) performance status, and chemotherapy regimen. Abbreviations: HR, hazard ratio; CI, confidence interval; ctDNA, circulating tumor DNA; VAF, variant allele frequency; CA19-9, carbohydrate antigen 19-9. Bold *p* values indicate statistical significance (*p* < 0.05).

## Data Availability

The datasets generated and analyzed during the current study are not publicly available due to patient privacy and confidentiality regarding clinical information, but are available from the corresponding authors upon reasonable request.

## Supplementary Materials

The following supporting information can be downloaded at: https://www.mdpi.com/article/10.3390/ijms27198668/s1.
