# Supplementary material for: Prognostic Value and Longitudinal Dynamics of Multigene Circulating Tumor DNA in Advanced Pancreatic Cancer Treated with 5-Fluorouracil-Based Chemotherapy
Source: Int J Mol Sci. 2026 Sep 28;27(19):8668. doi: 10.3390/ijms27198668 (PMC13664952; doi:10.3390/ijms27198668)
Supplement: Supplementary file 1 [file ijms-27-08668-s001.zip › ijms-4518819-supplementary.pdf]

Prognostic Value and Longitudinal Dynamics of Multigene Circulating Tumor DNA in Advanced Pancreatic Cancer Treated with 5-Fluorouracil-Based Chemotherapy

Supporting documents

Table S1. AlphaLiquid®100 panel of 118 genes

Figure S1. Distribution of *KRAS* mutation subtypes and clinical stage at diagnosis according to *KRAS* subtype.

Figure S2. Comparison of somatic mutation frequencies in pancreatic cancer between the NCC cohort and TCGA tumor tissue dataset

Figure S3. All mutation level VAF values per patient

Figure S4. Patient-level longitudinal trajectories of Top4 VAF during 5-FU-based chemotherapy.

Figure S5. Distribution of Top4 VAF percentage change from baseline by RECIST response category at FU1 and FU2.

Figure S6. Longitudinal ctDNA detection-state transitions during 5-FU-based chemotherapy and two representative patient-level examples.

Table S1. AlphaLiquid®100 panel of 118 genes

ABL1	CD274	FGFR3*	MAP2K1	NPM1	RHEB	U2AF1
AKT1	CDH1	FLT3	MAP2K2	NRAS	RHOA	UGT1A1
AKT2	CDK4	GATA3	MAPK1	NTRK1*	RIT1	VHL
ALK*	CDK6	GNA11	MAPK3	NTRK2*	RNF43	BARD1
APC	CDKN2A	GNAQ	MDM2	NTRK3	ROS1*	BCR**
AR	CEBPA	GNAS	MET	PDCD1LG2	RUNX1	BRIP1
ARAF	CSF1R	HRAS	MLH1	PDGFRA	SETD2	CDK12
ARID1A	CTNNB1	IDH1	MPL	PDGFRB	SMAD4	CHEK1
ATM	DDR2	IDH2	MSH2	PIK3CA	SMO	CHEK2
BRAF*	DPYD	IGF1R	MSH6	PIK3R1	STAG2	FANCL
BRCA1	EGFR*	JAK2	MTOR	PMS2	STK11	PALB2
BRCA2	ERBB2	JAK3	MYC	PPP2R1A	TCF7L2	PPP2R2A
BTK	ERBB3	KDM6A	MYCN	PTEN	TERT	RAD51B
CBL	ESR1	KDR	NF1	PTPN11	TOP2A	RAD51C
CCND1	FBXW7	KEAP1	NF2	RAF1	TP53	RAD51D
CCND2	FGFR1	KIT	NFE2L2	RB1	TSC1	RAD54L
CCNE1	FGFR2*	KRAS	NOTCH1	RET*	TSC2	

SNV, Indel, CNV 117 genes; * Fusion 10 genes; ** Intron region-only 1 gene; HRR gene 15 genes. SNV, single-nucleotide variant; Indel, insertion/deletion; CNV, copy number variation; HRR, homologous recombination repair.

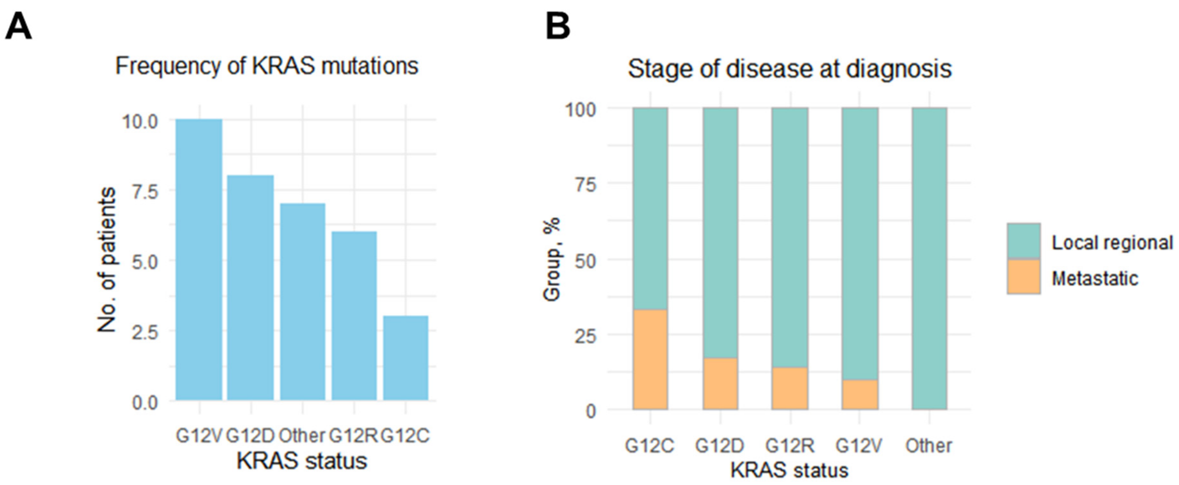

Figure S1. Distribution of *KRAS* mutation subtypes and clinical stage at diagnosis according to KRAS subtype. (A) Frequency of *KRAS* mutation subtypes detected in plasma ctDNA. G12V and G12D were the most common mutations observed, followed by other variants such as G12R and G12C. (B) Stage of disease at diagnosis (local/regional vs. metastatic) according to *KRAS* mutation subtype. While most patients across all *KRAS* subtypes were diagnosed at the local/regional stage, a higher proportion of patients with the G12C mutation presented with metastatic disease compared to other subtypes.

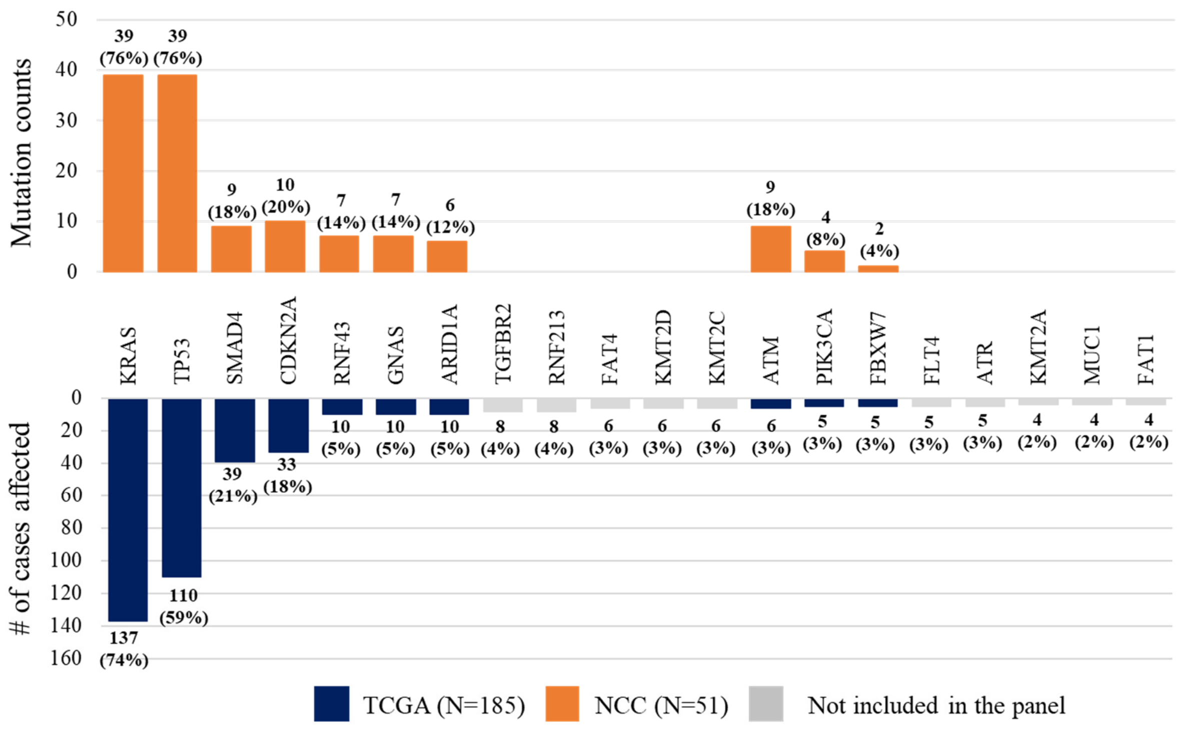

Figure S2. Comparison of somatic mutation frequencies in pancreatic cancer between the NCC cohort (n = 51, orange) and TCGA tumor tissue dataset (n = 185, navy). *KRAS* and *TP53* were the most frequently mutated genes in both cohorts. While most frequently altered genes overlapped between the two datasets, certain genes (e.g., *TGFBR2,* RNF213, *KMT2D*) detected in TCGA were not included in the AlphaLiquid100 panel (gray).

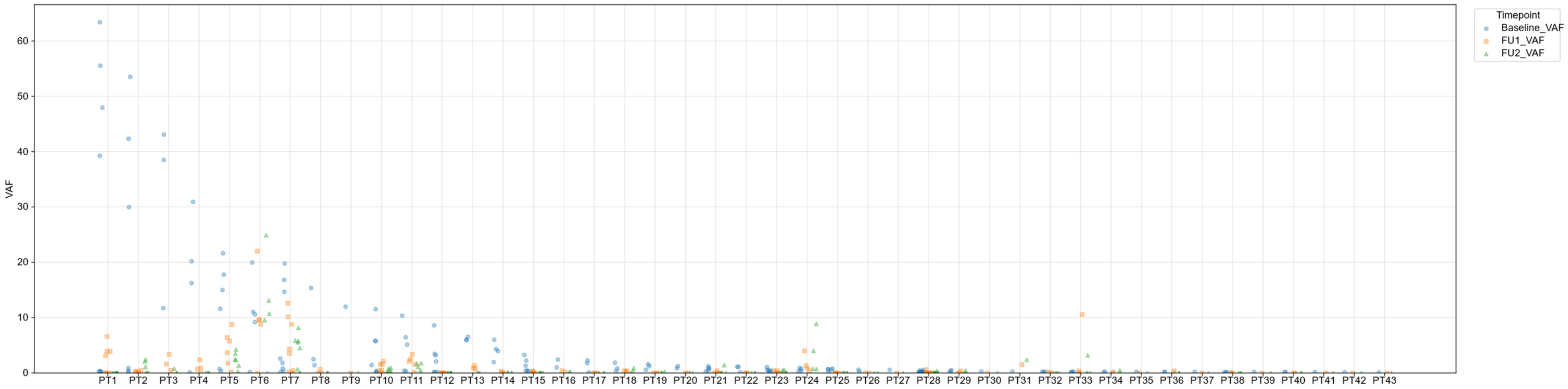

Figure S3. All mutation level VAF values per patient. Scatter plots show mutation-level VAF (%) for each patient at baseline (circles), first follow-up (FU1; squares), and second follow-up (FU2; triangles). Each point represents an individual somatic variant detected in a given sample. Patients on the x-axis are ordered by the maximum baseline VAF (descending)

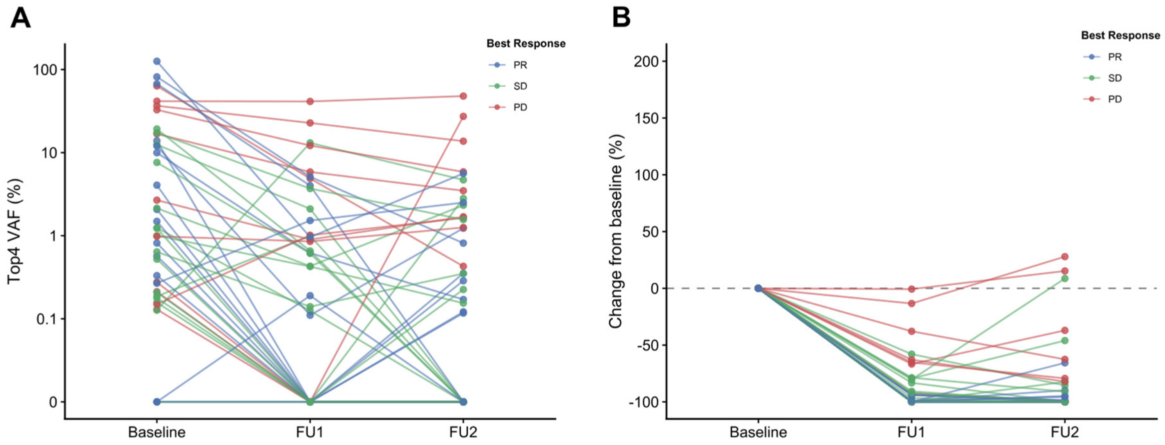

Figure S4. Patient-level longitudinal trajectories of Top4 VAF during 5-FU-based chemotherapy. (A) Spider plot showing absolute Top4 VAF (%) for each patient at baseline, FU1, and FU2. Each line represents one patient, and colors denote RECIST best response (PR, SD, PD). (B) Spider plot showing percent change in Top4 VAF from baseline (%) at FU1 and FU2. The horizontal dashed line indicates no change (0%); values below 0% indicate a decrease (molecular response) and values above 0% indicate an increase. Colors denote RECIST best response.

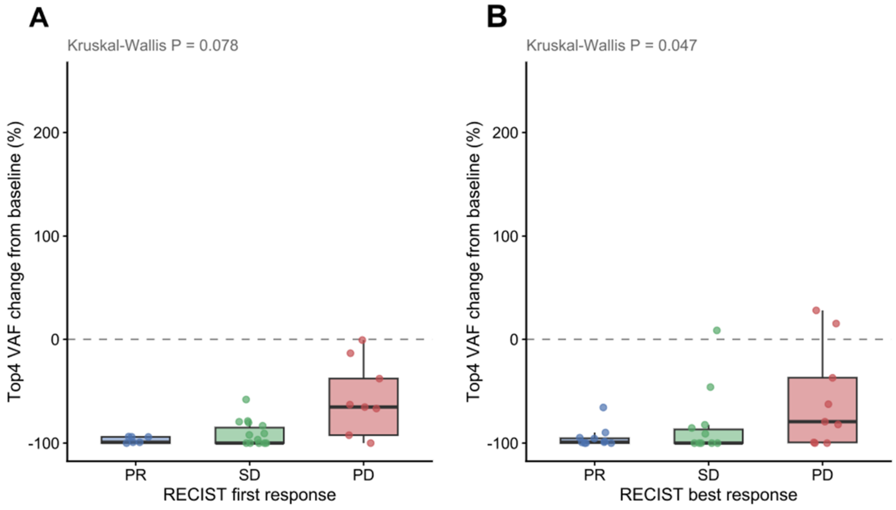

Figure S5. Distribution of Top4 VAF percentage change from baseline by RECIST response category at FU1 and FU2.

(A) Box-and-jitter plot of Top4 VAF percent change from baseline at FU1 (%) stratified by RECIST first response; Kruskal-Wallis *P* value is shown. The dashed line indicates 0% (no change). (B) Box-and-jitter plot of Top4 VAF percent change from baseline at FU2 (%) stratified by RECIST best response; Kruskal-Wallis *P* value is shown. In (A-B), boxes indicate median and interquartile range; whiskers indicate the distribution as defined in the plotting scheme; points represent individual patients. Negative values indicate VAF reduction from baseline.

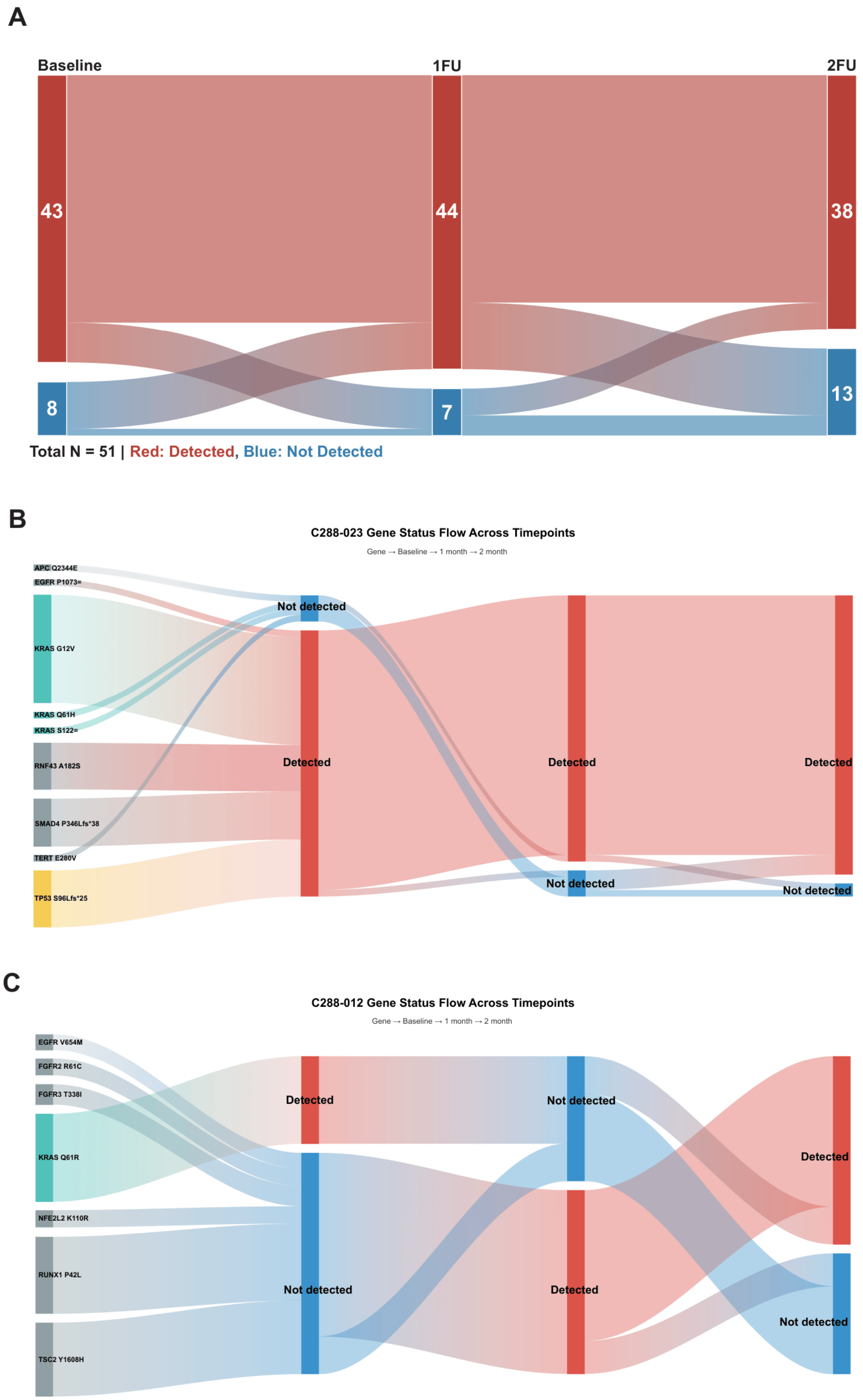
A
Baseline
1FU
2FU
43
44
38
8
7
13
Total N = 51 | Red: Detected, Blue: Not Detected
B
C288-023 Gene Status Flow Across Timepoints
Gene → Baseline → 1 month → 2 month
APC Q2344E
EGFR P1073=
KRAS G12V
KRAS Q61H
KRAS S122=
RNF43 A182S
SMAD4 P346Lfs*38
TERT E280V
TP53 S96Lfs*25
Not detected
Detected
Detected
Not detected
Detected
Not detected
C
C288-012 Gene Status Flow Across Timepoints
Gene → Baseline → 1 month → 2 month
EGFR V654M
FGFR2 R61C
FGFR3 T338I
KRAS Q61R
NFE2L2 K110R
RUNX1 P42L
TSC2 Y1608H
Detected
Not detected
Not detected
Detected
Detected
Not detected

Figure S6. Longitudinal ctDNA detection-state transitions during 5-FU-based chemotherapy and two representative patient-level examples. (A) Alluvial plot summarizing ctDNA detection status across three sampling time points (baseline, FU1, and FU2) in the overall cohort (n = 51). Stream widths are proportional to the number of patients transitioning between "Detected" and "Not detected" states, with counts displayed within each state. (B) Patient-level alluvial plot illustrating longitudinal detection dynamics of individual alterations in a representative patient with an unfavorable clinical course. (C) Patient-level alluvial plot illustrating longitudinal detection dynamics of individual alterations in a representative patient with a favorable clinical course. In panels (B-C), each ribbon represents one tracked alteration and highlights within-patient heterogeneity, including persistent alterations, transient loss of detectability, and newly emergent alterations during follow-up
